# CT perfusion in hyper-acute ischemic stroke: the acid test for COVID-19 fear

**DOI:** 10.1007/s00234-021-02639-5

**Published:** 2021-02-02

**Authors:** Giovanni Furlanis, Miloš Ajčević, Ilario Scali, Alex Buoite Stella, Sasha Olivo, Carlo Lugnan, Paola Caruso, Roberta Antea Pozzi Mucelli, Agostino Accardo, Maria Assunta Cova, Marcello Naccarato, Paolo Manganotti

**Affiliations:** 1 Clinical Unit of Neurology, Department of Medicine, Surgery and Health Sciences, University Hospital and Health Services of Trieste – ASUGI, University of Trieste, Strada di Fiume, 447, 34149 Trieste, Italy; 2 Department of Engineering and Architecture, University of Trieste, Via A. Valerio, 10, 34127 Trieste, Italy; 3 Radiology Unit, Department of Medicine, Surgery and Health Sciences, University Hospital and Health Services of Trieste – ASUGI, University of Trieste, Strada di Fiume, 447, 34149 Trieste, Italy

**Keywords:** Stroke, COVID, Coronavirus, CT perfusion, Image processing, Perfusion pattern

## Abstract

**Purpose:**

The fear of COVID-19 infection may discourage patients from going to the hospital even in case of sudden onset of disabling symptoms. There is growing evidence of the reduction of stroke admissions and higher prevalence of severe clinical presentation. Yet, no studies have investigated the perfusion pattern of acute strokes admitted during the lockdown. We aimed to evaluate the effects of the COVID-19 pandemic on hyper-acute stroke CT perfusion (CTP) pattern during the first months of the pandemic in Italy.

**Methods:**

In this retrospective observational study, we analyzed CTP images and clinical data of ischemic stroke patients admitted between 9 March and 2 June 2020 that underwent CTP (*n* = 30), to compare ischemic volumes and clinical features with stroke patients admitted during the same period in 2019 (*n* = 51). In particular, CTP images were processed to calculate total hypoperfused volumes, core volumes, and mismatch. The final infarct volumes were calculated on follow-up CT.

**Results:**

Significantly higher total CTP hypoperfused volume (83.3 vs 18.5 ml, *p* = 0.003), core volume (27.8 vs 1.0 ml, *p* < 0.001), and unfavorable mismatch (0.51 vs 0.91, *p* < 0.001) were found during the COVID-19 period compared to no-COVID-19 one. The more unfavorable perfusion pattern at admission resulted in higher infarct volume on follow-up CT during COVID-19 (35.5 vs 3.0 ml, *p* < 0.001). During lockdown, a reduction of stroke admissions (− 37%) and a higher prevalence of severe clinical presentation (NIHSS ≥ 10; 53% vs 36%, *p* = 0.029) were observed.

**Conclusion:**

The results of CTP analysis provided a better insight in the higher prevalence of major severity stroke patients during the COVID-19 period.

## Introduction

SARS-CoV2 is a novel coronavirus identified as the cause of coronavirus disease 2019 (COVID-19) that began in Wuhan, China, in late 2019 spreading worldwide leading to a WHO declaration of world pandemic on 11 March 2020 [1]. The COVID-19 pandemic caused lockdown in a large part of the world, entailing movement restriction except in case of necessity, work, and health circumstances. In these circumstances, healthcare systems have been focused primarily on COVID-19 management and the citizens on COVID-19 symptoms. Further, the fear for infection discourages patients from going to the hospital even in case of sudden onset of neurological and disabling symptoms. A recent study observed a reduction of 45% of total admissions in a Hub Stroke Unit compared to the same period in 2019 and a higher prevalence of severe stroke (NIHSS > 10) at admission during the COVID-19 pandemic, probably due to a lower number of Emergency Department admissions of transient ischemic attack (TIA) and minor stroke patients [2].

Neuroimaging is a vital component in the objective assessment of patients with symptoms compatible with acute ischemic stroke [3–6]. In the hyperacute phase, decision-making is always more based on the *tissue clock* approach instead of on the *time clock* approach. Perfusion-based neuroimaging, such as CT perfusion (CTP), can identify patients who can most benefit from the recanalization treatment by identifying irreversible infarct core and the ischemic salvageable penumbra area [7], which is especially useful in extended time windows patients or wake-up stroke [8–12].

The severity of neurological deficits in terms of NIHSS at admission was found to be highly correlated with the size of hypoperfused tissue assessed by CTP processing [5] and by MR perfusion imaging (MRP)[13]. Lesion volumes assessed by MRP in acute stroke were demonstrated to be highly associated with the NIHSS score at 24 h [14]. In addition, the functional outcome is related to the final infarct volume and also depends on lesion location [15, 16].

Recently, some reports highlighted the trend of the reduction of stroke admissions and higher prevalence of severe clinical presentation during the COVID-19 pandemic [2, 17, 18]. There is a growing interest for the use of CTP data in acute stroke research; however, no studies have been conducted to investigate the perfusion pattern of acute stroke patients admitted during lockdown.

To evaluate the effects of the COVID-19 pandemic on stroke CTP pattern, this study compared CTP features and clinical data of hyper-acute ischemic stroke patients admitted between 9 March 2020 (start of Italy lockdown) and 2 June 2020 with stroke patients admitted during the same period in 2019.

## Methods

### Study population

In this retrospective observational study, we processed and analyzed CT perfusion and clinical data of patients with hyper-acute ischemic stroke admitted to the Stroke Unit of the University Medical Hospital of Trieste (Italy) between 9 March 2020 (start of Italy lockdown) and 2 June 2020 (end of mobility restriction period in Italy*)* (*COVID-19 period*) and compared perfusion patterns and clinical features with patients admitted during the same period in 2019 (*no-COVID period*). We included hyper-acute ischemic stroke patients admitted within 4.5 h from symptoms onset, stroke patients with suspect of large vessel occlusion (LVO) within 6 h from symptoms onset, and stroke patients with undetermined onset (SUSO) who underwent multiparametric CT recordings as per internal management protocol for all “code stroke” patients. All included patients underwent multiparametric CT assessment, consisting of non-enhanced CT (NECT), CT angiography (CTA), and CTP. No age limit was applied and both genders were included. Hemorrhagic stroke and patients with transitory ischemic attack (TIA) were excluded. Stroke mimics were excluded by a complete diagnostic workup including clinical and MRI assessment to confirm the absence of ischemic lesion. A trained neurologist performed stroke severity evaluation using the NIHSS at admission and evaluated the anamnestic level of independence with the mRS.

Patients eligible for thrombolysis were treated with intravenous rtPA (0.9 mg/kg of body weight, maximum of 90 mg, infused over 60 min with 10% of the total dose administered as an initial intravenous bolus over 1 min). Endovascular thrombectomy (EVT) was performed in eligible patients with intracranial large vessel occlusions (M1, M2, A1, ICA, and basilar artery) according to standard clinical practice [19]. Infarct final lesion was evaluated by follow-up NECT (at 24 to 48 h). A common neurologic stroke workup including assessment of stroke risk factors, ECG, intracranial and extracranial ecocolordoppler, echocardiography, and Holter electrocardiography or telemetric ECG monitoring. ECASS - European-Australian Cooperative Acute Stroke Study 3 criteria were used to define symptomatic hemorrhage (sICH) [20].

The following data of included patients were collected: (1) demographic details (e.g., age, sex); (2) stroke etiology by TOAST classification [21]; (3) stroke syndrome by Bamford classification (total anterior circulation infarct (TACI), partial anterior circulation infarct (PACI), lacunar stroke (LACI) posterior circulation infarct (POCI)); (4) National Institutes of Health Stroke Scale (NIHSS) score at admission and on the 7th day or before in case of discharge; (5) premorbid modified Rankin score (mRS) and mRS at discharge; (6) stroke risk factors (arterial hypertension, diabetes mellitus, dyslipidemia, ischemic cardiopathy, atrial fibrillation); (7) NECT (ASPECTscore) and final infarct volume at follow-up NECT; (8) large vessel occlusion; (9) total hypoperfused CTP volume, core CTP volume, mismatch CTP; (10) symptomatic intracerebral hemorrhage (sICH); (11) rTPA therapy and/or EVT; (12) time from alert-to-admission; (13) time from alert-to-treatment; (14) time from alert-to-multiparametric CT scan; and (15) length of hospitalization and destination at discharge.

All procedures performed in the study were approved by the local ethics committee in accordance with the ethical standards of the institutional research committee and with the 1964 Helsinki declaration and its later amendments or comparable ethical standards. Informed consent was obtained from all individual participants included in the study.

### CT and CTP acquisition and processing

All CT images were acquired by using a 256-slice Philips Brilliance iCT scanner (Philips Healthcare, Best, The Netherlands) at 80 kVp and 150–200 mAs. CTP data were processed by using Extended Brilliance Workstation v 4.5 (Philips Medical Systems, Best, Netherlands) and a home-made script created in Matlab (MathWorks Inc., Natick, MA). Perfusion maps, namely mean transit time (MTT), cerebral blood volume (CBV), and cerebral blood flow (CBF) were calculated. MTT maps were estimated via a closed-form deconvolution operation using the time/concentration curve of a particular voxel and the arterial input function obtained from source images. The CBV map was calculated from the area under the time/concentration curves and CBF as ratio between CBV and MTT. Core and hypoperfused penumbra areas were automatically identified by applying thresholds defined by Wintermark et al. [22]. In particular, the infarcted core areas were identified as MTT > 145% of the contralateral healthy area and CBV < 2.0 ml/100 g, while penumbra voxels were identified as MTT higher than 145% of the contralateral healthy area and CBV > 2.0 ml/100 g. The ischemic volumes were calculated after the artifact removal by integration of identified areas as previously described [5, 12].

Final infarct lesion volume was calculated on follow-up NECT for all patients by a semi-automatic algorithm for segmentation, implemented in Matlab (MathWorks, Natick, MA), based on seed-based region growing algorithm, and also supported by additional manual outlining. Two independent neurologists checked the results of this semi-automatic process.

### Statistical analysis

We performed all statistical analysis using SPSS Statistics 23 (IBM, Armonk/NY, USA). Subgroup analysis and data presentation was proposed for 2019 and 2020 patients. Kolmogorov-Smirnov test was adopted to evaluate the normal distribution of variables. Continuous variables with normal distribution are presented as mean and standard deviations (SDs), those with a skewed distribution as median and interquartile ranges (IQRs) indicating the 1st and 3rd quartile, and categorical variables as counts, percentages, and 95% confidence interval (*n*, %; 95% CI). Differences between the two groups were tested with parametric (independent samples *T* test), nonparametric tests (Mann-Whitney *U* test), and chi-square, as appropriate. Univariate linear regression (*B*, 95% CI) was performed to assess the association between the COVID-19 period and calculated neuroimaging features, while binary logistic regression (OR, 95% CI) was performed to assess the association between the COVID-19 period and large vessel occlusion. A level of *p* < 0.05 was regarded as statistically significant.

## Results

During the COVID-19 period, a total of 62 patients were admitted within 24 h from stroke onset to the Stroke Unit compared to the 98 patients admitted in the same period in 2019 (− 37%). In patients admitted within the 24 h window, a slightly higher NIHSS at admission was observed in the COVID-19 period (median: 10; 4–19 vs 6; 4–17), although non statistically significant (*p* = 0.135). Prevalence of patients with NIHSS at admission ≥ 10 was significantly higher during the COVID-19 period (*n* = 33, 53%; 41–66% vs *n* = 35, 36%; 26–44%, *p* = 0.029).

In the COVID-19 period, as many as 30 (16F/14M, age median: 76 years) out of 62 admitted patients underwent multiparametric CT as “code stroke” patients fulfilling inclusion criteria, while 51 (22F/29M, age median: 76 years) out of 98 in the corresponding no-COVID-19 period. All patients admitted to our Stroke Unit in the COVID-19 period underwent nasopharyngeal swab: all tests were negative for SARS-CoV-2. The demographic, risk factors as well as clinical outcome of COVID-19 period and no-COVID-19 period included patients are presented in Table 1.

**Table 1:** Demographics and clinical data of patients admitted in COVID-19 period (2020) versus no-COVID-19 period (2019). Data are presented as medians (IQR) and frequencies

Personal characteristics	COVID-19 (*n* = 30)	No-COVID-19 (*n* = 51)	*p *value
Age [y]	76 (68–82)	76 (72–83)	0.618
Female:Male	16:14	22:29	0.375
SUSO (%)	9 (30%)	9 (18%)	0.197
Treatment (%)			
rTPA (%)	21 (70%)	37 (73%)	0.806
EVT (%)	7 (23%)	10 (20%)	0.681
NIHSS at baseline	11 (4–17)	6 (4–15)	0.317
NIHSS ≥ 10 (%)	17 (57%)	16 (31%)	**0.025**
NIHSS at discharge	5 (1–21)	1 (0–7)	0.071
mRS 0–2 anamnestic (%)	29 (97%)	48 (94%)	0.609
mRS 0–2 at discharge (%)	12 (40%)	29 (57%)	0.143
Intrahospital mortality (%)	4 (13%)	6 (12%)	0.836
Bamford Classification (%)			0.510
TACI	12 (40%)	14 (27%)	
PACI	10 (33%)	18 (35%)	
LACI	4 (13%)	13 (25%)	
POCI	4 (13%)	6 (12%)	
sICH	3 (10%)	2 (4%)	0.272
TOAST (%)			0.468
Atherothrombotic	2 (7%)	6 (12%)	
Small vessel	2 (7%)	10 (20%)	
Cardioembolic	16 (53%)	21 (41%)	
Cryptogenic	9 (30%)	12 (24%)	
Other	1 (3%)	2 (4%)	
Risk factors (%)			
Hypertension	25 (83%)	41 (80%)	0.742
Diabetes	12 (40%)	18 (35%)	0.672
Dyslipidemia	18 (60%)	33 (65%)	0.672
Atrial fibrillation	14 (47%)	20 (39%)	0.512
Ischemic cardiomyopathy	7 (23%)	16 (31%)	0.438
Length of hospitalization (%)	8 (5–10)	6 (4–17)	0.680
Destination at discharge (%)			0.552
Home	11 (42%)	21 (49%)	
Rehabilitation	5 (19%)	6 (14%)	
Neuro spoke	7 (27%)	10 (23%)	
Other	3 (10%)	6 (14%)	

Notes: Participants’ reported age (y), sex (female:male), stroke of unknown symptom onset (SUSO), thrombolytic therapy (rTPA), endovascular thrombectomy (EVT), NIHSS at admission (number of patients with NIHSS ≥ 10) and NIHSS at discharge, anamnestic modified Rankin scale (mRS) and mRS at discharge, Intra-hospital mortality, Bamford stroke syndrome classification (total anterior circulation infarct, TACI; partial anterior circulation infarct, PACI; lacunar stroke, LACI; posterior circulation infarct, POCI), stroke subtype classification at discharge classified by Trial of ORG 10172 in Acute Stroke Treatment (TOAST): atherothrombotic, small vessel disease, cardioembolic, cryptogenic, other cause, history of hypertension (HTN), diabetes (DM), dyslipidemia, atrial fibrillation (AF), ischemic cardiomyopathy (ICM), length of hospitalization, destination at discharge (home, rehabilitation department, neurology department in spoke hospital, other department). Results are summarized for patients admitted in our Stroke Unit in COVID-19 period (9 March–2 June 2020) and in no-COVID-19 period (9 March–2 June 2019). Bold values for significance value for intergroup comparison (*p* < 0.05—highlighted in bold)

There were no differences in alert-to-admission (108 min vs 89 min, ns), alert-to-CT (155 min vs 150 min, ns), and in alert-to-treatment times (180 min vs 176 min, ns), between the two groups. The percentage of patients who underwent rTPA treatment and patients underwent EVT was similar between the two groups (Table 1). A higher prevalence of severe stroke (NIHSS ≥ 10) was found in COVID-19 period compared to 2019 (*n* = 17, 57%; 39–74% vs *n* = 16, 31%; 19–44%, respectively; *p* = 0.025). A slightly higher prevalence of SUSO was observed in 2020 (*n* = 9, 30%, 14–46%) compared to 2019 (*n* = 9, 18%, 7–28%). No difference in prevalence of risk factors like arterial hypertension, diabetes, dyslipidemia, and atrial fibrillation between the two periods was observed. In addition, a slightly higher trend of prevalence of TACI was found in 2020 patients (*n* = 12, 40%; 25–56%) than 2019 patients (*n* = 14, 27%; 15–40%).

**Fig. 1:**
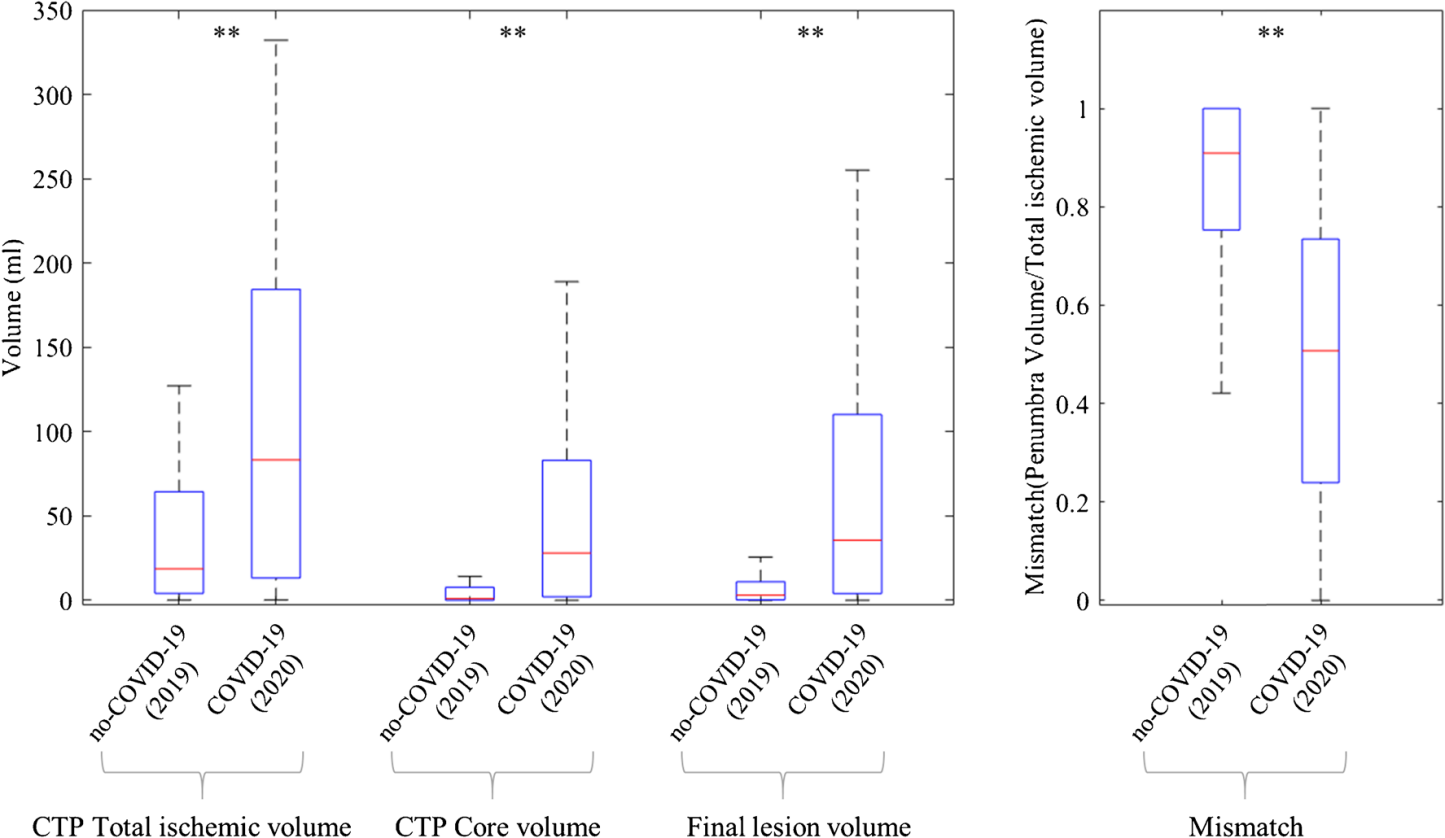
Comparison of CT perfusion (CTP) features at admission and final infarct volume on follow-up CT between COVID-19 and no-COVID-19 periods. Box and whisker plot (medians, IQR values, range). Significant differences: *p < 0.05, **p < 0.01

Table 2 reports the neuroimaging findings, namely ASPECTs assessed on NECT, large vessel occlusion on CTA as well as calculated CTP features. Figure 1 reports comparison of CTP features at admission and final infarct volume on follow-up CT between COVID-19 period and no-COVID 19 period.

**Table 2:** Neuroimaging data of patients admitted in COVID-19 period (2020) versus no-COVID-19 period (2019)

Neuroradiological assessment	COVID-19 (*n* = 30)	No-COVID-19 (*n* = 51)	*p* value	Regression	
				*B*/OR (95% CI)	*p* value
NECT					
ASPECTs	10 (9–10)	10 (9–10)	**0.026**	− 0.46 (− 1.00–0.80)	0.094
CTA					
Large vessel occlusion n (%; 95% CI)	16 (53.3%; 35.5–71.2)	17 (33.3%; 20.4–46.3)	0.077	2.84 (1.08–7.47)	**0.034**
CTP					
CTP total hypoperfused volume [ml]	83.3 (22.0–187.8)	18.5 (3.5–77.2)	**0.003**	62.94 (29.96–95.91)	**<0.001**
CTP core volume [ml]	27.8 (0–120.2)	1.0 (0–15.9)	**<0.001**	52.20 (29.37–76.03)	**<0.001**
CTP mismatch	0.51 (0.23–0.94)	0.91 (0.74–1)	**<0.001**	− 0.29 (− 0.41–0.16)	**<0.001**
Final infarct volume [ml]	35.5 (3.0–150.8)	3.0 (0–28.3)	**<0.001**	58.24 (29.96–86.51)	**<0.001**

Note: Data are presented as medians (IQR) and frequencies (%; 95% CI—confidence interval). Non-enhanced computed tomography (NECT) and Alberta Stroke Program Early CT Score (ASPECTS); CT angiography (CTA) and large vessel occlusion (M1, M2, A1, intracranial ICA, and basilar artery). CT perfusion (CTP) features. Significant differences (*p* < 0.05) are highlighted in bold. Univariate linear regression between COVID-19 period and calculated neuroimaging features: *B* coefficient (95% CI). Binary logistic regression between the COVID-19 period and large vessel occlusion: odds ratio OR (95% CI); all included patents during the COVID-19 period (*n* = 30) and no-COVID-19 period (*n* = 51) underwent CTP and follow-up CT and were included in the analysis

In the COVID-19 period, acute ischemic stroke presented a significantly higher total CTP hypoperfused volume (83.3 vs 18.5 ml, *p* = 0.003) and higher core CTP volume (27.8 vs 1.0 ml, *p* < 0.001), as well as a lower prevalence of penumbra compared to core yeling a lower CTP mismatch (0.51 vs 0.91, *p* < 0.001). ASPECT was significantly lower in the COVID-19 period compared to no-COVID-19 period (*p* = 0.026). A slightly higher prevalence of large vessel occlusion in COVID-19 period was observed, although none statistically significant (53.3%; 35.5–71.2 vs 33.3%; 20.4–46.3, *p* value = 0.077). These results were also supported by regression analysis reported in Table 2.

Regarding the morphological outcome in terms of extent of necrotic tissue on follow-up NECT, the final infarct volume was significantly higher in the COVID-19 period compared to the no-COVID-19 (35.5 vs 3 ml, *p* < 0.001). Furthermore, a slightly higher NIHSS at discharge was observed in the COVID-19 period compared to the no-COVID-19, although not statistically significant (median NIHSS = 5 vs 1, *p* value = 0.071).

## Discussion

In the first months of the COVID-19 pandemic, the main focus of health authorities and healthcare providers was the SARS-CoV2 infected population. This had a relevant impact on treatment of time-dependent pathologies such as stroke and acute coronary syndrome [2, 23]. A significant reduction of stroke admissions and a higher prevalence of severe clinical presentation were observed worldwide due to the spread of COVID-19 outbreaks [2]. This study aimed at deepening the analysis of tissue-based stroke patient’s characteristics during the COVID-19 pandemic compared to the reference period.

**Fig. 2:**
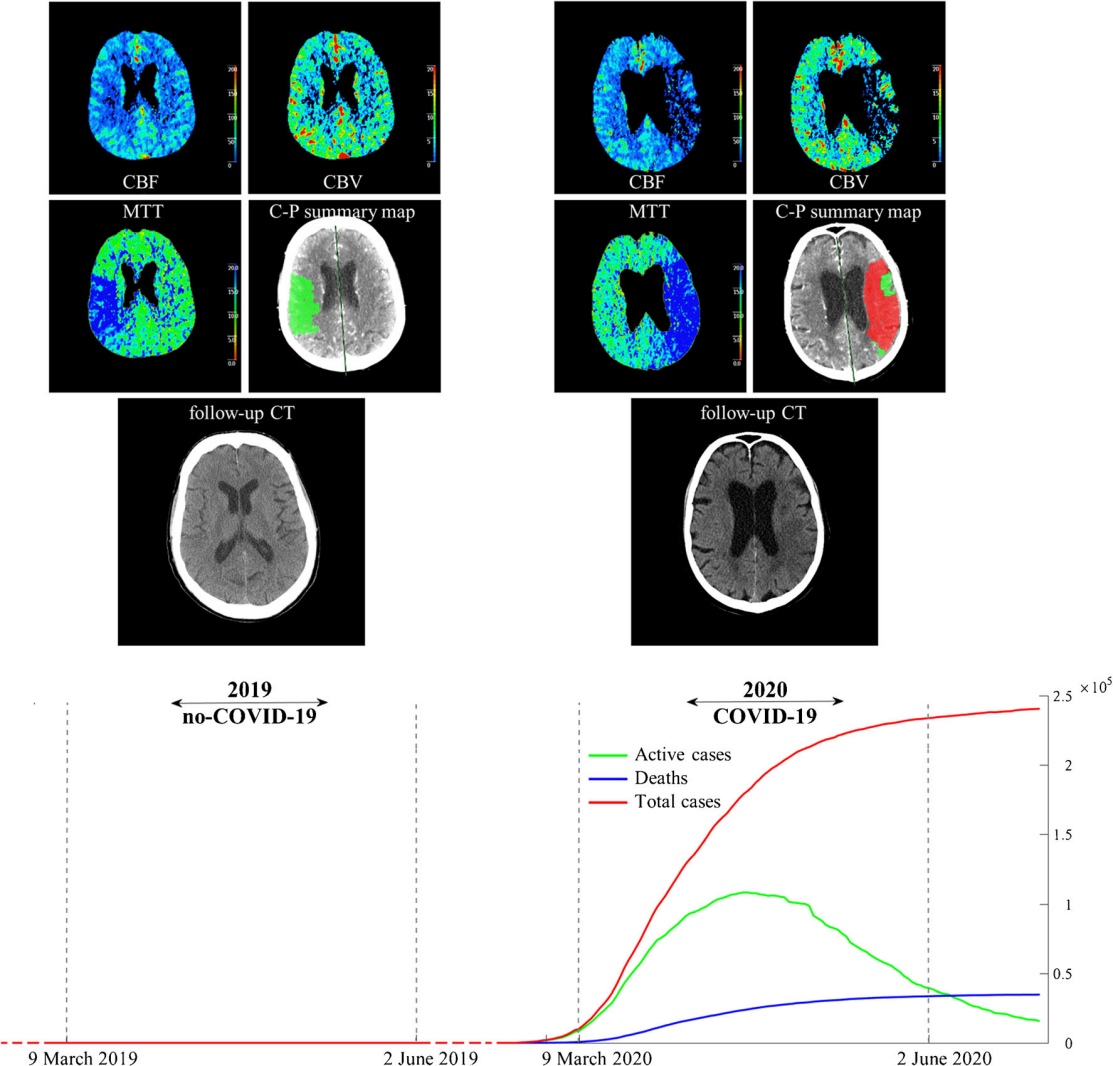
Comparison between CT perfusion and follow-up CT during COVID-19 and no-COVID-19 periods. Epidemic outbreak in Italy (data from Italian Ministry of Health daily official report, see http://www.salute.gov.it/portale/home.html). During the study period (9 March–2 June 2020) a progressive increase of confirmed and active COVID-19 cases in Italy and related deaths. The active cases reached a plateau in the first days of May. The exemplifying perfusion patterns at admission as well as follow-up CT lesion extension are reported in the figure for both study periods (no-COVID-19. left panel; COVID-19, right panel). A higher total CTP hypoperfused volumes, CTP core volumes and unfavorable mismatch were observed in the COVID-19 period compared to the no-COVID-19. The unfavorable perfusion pattern at admission resulted in a large final infarct lesion in follow-up CT. During lockdown a reduction of stroke admissions (− 37%) and a higher prevalence of severe clinical presentation in terms of NIHSS ≥ 10 was observed. MTT, mean transit time; CBV, cerebral blood volume; CBF, cerebral blood flow; Core-Penumbra summary map, Core (red)/Penumbra (green) summary map. CT follow-up with final ischemic lesion

The main finding of this study is a significantly higher perfusion deficit in terms of total CTP hypoperfused volume and CTP core volume, as well as significantly unfavorable penumbra to core mismatch in the COVID-19 period compared to the no-COVID-19. In particular, a significantly higher total CTP hypoperfused volume (83.3 vs 18.5 ml) and CTP irreversible core volume (27.8 vs 1.0 ml) were detected, resulting in a significantly lower mismatch, i.e., percentage of penumbra of total hypoperfused volume (0.51 vs 0.91). The main findings are summarized in Fig. 2.

These results obtained by perfusion imaging offer a better insight in the differences in stroke clinical presentation observed as NIHSS score (median NIHSS = 11 vs 6) and a significantly higher prevalence of severe stroke presentations with a NIHSS ≥ 10 (57% vs 31%, *p* = 0.025). The prevalence of severe stroke (NIHSS ≥ 10) reflects the high CTP hypoperfused volumes observed in the COVID-19 period. This link between stroke severity and perfusion deficit is consistent with previous findings regarding the high correlation between the baseline NIHSS score and the ischemic volume in CTP imaging in the acute stroke phase. In a recent study, we found a significantly strong correlation (*r* = 0.82, *p* = < 0.0001) between total hypoperfused CTP volume and NIHSS at admission in 106 hyperacute ischemic stroke patients [5]. Low NIHSS values mainly corresponded to low volumes and, vice versa, high NIHSS values corresponded to high volumes.

In the COVID-19 period, we observed a reduction of 37% of total admissions in our Hub Stroke Unit and a prevalence of very disabling stroke with NIHSS ≥ 10 compared to the same period in 2019. This may be explained with the lower number of emergency department admissions of transient ischemic attack (TIA) and minor strokes related to the diffused fear of going to the hospital during the pandemic when showing mild or transient stroke symptoms. Indeed, minor stroke and TIA patients are characterized by minor CTP hypoperfused volumes [5], while severe stroke patients characterized by disabling symptoms and a large hypoperfused ischemic volume could not refrain from calling emergency.

The significantly higher prevalence of core presence in total hypoperfused volume during the COVID-19 pandemic could be a sign that the patients may have waited until the last minute to call the alert. Indeed, we also observed a trend of increase of SUSO patients. In addition, a slight prevalence of cardioembolic strokes, characterized by severe presentation and absence of collaterals development [24], and patients with comorbidities could contribute to the worse perfusion pattern.

The fear of in-hospital SARS-CoV2 infection created anxiety in the general population forcing many patients with acute and disabling diseases to avoid going to the hospital. Our neuroimaging and clinical results support the hypothesis of perceived fear taking into account that our served area (Friuli Venezia Giulia region) population characterized by a high prevalence of elderly with polymorbidities [25], did not show the same high COVID-19 spread as the other northern Italy regions.

Higher unfavorable perfusion pattern at admission, taking into account the same percentage of rTPA/EVT treated patients, supports the finding of higher infarct volume on follow-up NECT during COVID-19 pandemic (35.5 vs 3.0 ml, *p* < 0.001). The results of our study showed that CTP hypoperfused parameters are correlated with final infarct volume, which is in line with previous studies based on CTP [26, 27]. Shankar et al. (2016) found that CTP infarct core was the major predictor of final infarct volume and, at the same time, total hypoperfused volume measured by CBF was also correlated with the final lesion [26].

Advanced multimodal neuroimaging plays an increasingly relevant role in the management of patients with stroke-related symptoms [4, 6, 7, 11, 12, 28, 29]. Using CTP to identify irreversible infarct core and the ischemic salvageable penumbra area in a quick and accurate manner allows stroke emergency management based on the tissue-clock approach instead of on the time-clock approach. CTP is notably a fast imaging technique, also characterized by high sensitivity (80%) and specificity (95%) [30]. In addition, CTP perfusion data may provide useful information as a complementary asset to understand the pathophysiological mechanisms underlying the clinical phenomenon.

Our study had some limitations. The data describe a single-center investigation of a limited sample size. Moreover, although the included patients were consecutive, we conducted a retrospective observational study. In this study, we investigated all hyper-acute stroke patients admitted as “code stroke” patients (within 4.5 h from stroke onset, within 6 h from stroke onset in case of suspect LVO, or SUSO) who underwent multimodal CT assessment as per regional clinical care integrated pathway for acute stroke. Patients alerting the emergency services after 4.5 h (or after 6 h in case of suspect of LVO) were not labeled as “code stroke” and consequently did not undergo CT perfusion assessment. Thus, such patients were not been included in this perfusional study. The results should be confirmed in a larger clinical study including CT perfusion assessment on patients admitted in a time window extended up to 24 h from onset. In addition, CT perfusion lower reliability in minor strokes (especially in lacunar etiology) should be always taken into account. Nevertheless, our study contributes to give a better insight on the COVID-19 emergency and lockdown impact on stroke pathway by implementing whole brain CTP processing in real-life acute stroke admitted patients.

## Conclusion

The results of this study highlighted the ischemic stroke CTP perfusion pattern as an acid test for the COVID-19 fear in acute ischemic stroke. During the COVID-19 pandemic, a higher percentage of severe strokes and concomitant higher hypoperfused volumes and an unfavorable mismatch were observed resulting in higher infarct brain lesion. These tissue-evidenced findings deepened the analysis of the consequences on acute stroke pathway caused by the fear for the pandemic, while also pointing out the novel scenario that first-line neurologists have to face during the COVID-19 pandemic.
